# The second victim phenomenon in Japan: prevalence, impact, and associated factors— the JaSeVic study

**DOI:** 10.1186/s12913-026-14251-5

**Published:** 2026-02-25

**Authors:** Gen Aikawa, Misa Tomooka, Hideaki Sakuramoto, Yumi Matsumura, Takeshi Unoki, Akira Ouchi, Mitsuki Ikeda, Ayako Fukushima, Yuma Ota, Kentaro Kaneko

**Affiliations:** 1 College of Nursing, Kanto Gakuin University, Yokohama, Kanagawa Japan; 2 Department of Nursing, Faculty of Life Sciences, Kumamoto University, Kumamoto, Japan; 3 Division of Faculty Development, Nursing, Kindai University, Osaka, Japan; 4 Department of Patient Safety, Kyoto University Hospital, Kyoto, Japan; 5 Department of Acute and Critical Care Nursing, School of Nursing, Sapporo City University, Sapporo, Japan; 6 Department of Adult Health Nursing, College of Nursing, Ibaraki Christian University, Hitachi, Japan; 7 Department of Emergency and Critical Care Medicine, Faculty of Medicine, University of Tsukuba, Tsukuba, Ibaraki Japan; 8 Department of Nursing, Hokkaido University of Science, Sapporo, Japan; 9 Faculty of Healthcare, Division of Nursing, Tokyo Healthcare University, Higashigotanda, Tokyo Japan; 10 School of Nursing, Miyagi University, Kurokawa, Miyagi Japan

**Keywords:** Second victim, Patient safety incidents, Health personnel, Organizational support, Patient safety culture

## Abstract

**Background:**

Patient safety incidents (PSIs) not only affect patients but also cause psychological and physical distress in healthcare professionals, known as the second victim phenomenon (SVP). Understanding the prevalence, characteristics, and support needs related to SVP is essential to promote healthcare worker well-being and patient safety in Japan.

**Methods:**

The study conducted a web-based, cross-sectional survey in Japan that targeted experienced healthcare professionals on the mailing lists of academic societies or organizations. The survey assessed the types, prevalence, recovery time, impacts, and desired support related to SVP. Structural equation modeling (SEM) and mediation analysis were used to examine associations among organizational support, patient safety culture, second victim (SV) distress, and outcomes (turnover intentions, absenteeism, and resilience). Multiple regression analysis examined factors associated with SV distress.

**Results:**

A total of 884 participants responded, and 78.1% reported experiencing SVP. Individuals directly involved in PSIs reported higher levels of distress and perceived less institutional support than observers. Only 15.7% reported the presence of organizational support. Recovery exceeded one year in 13.9% of cases, and 9.7% reported persistent symptoms. Organizational support and a positive patient safety culture were associated with lower SV distress, turnover intentions, and absenteeism, and with higher resilience (all *p* < 0.05). Mediation analysis indicated indirect associations through reduced SV distress. The most significant factors associated with lower SV distress were nonpunitive responses to errors (*B* = − 0.132, 95% CI [− 0.195, − 0.070]) and colleague support (*B* = − 0.582, 95% CI [− 0.659, − 0.505]).

**Conclusions:**

The findings indicate that SVP is common and often prolonged among healthcare professionals and that those directly involved in PSIs report greater vulnerability. Organizational support, including colleague support, and a nonpunitive patient safety culture were associated with lower SV distress and lower turnover intentions.

**Supplementary Information:**

The online version contains supplementary material available at 10.1186/s12913-026-14251-5.

## Background

Patient safety incidents (PSIs) are defined as “any deviation from usual medical care that either causes an injury to the patient or poses a risk of harm, including errors, preventable adverse events, and hazards” [1]. This definition includes not only adverse events that result in patient harm but also ‘near misses’ that are intercepted before reaching the patient and ‘hazards’ that represent potential dangers. While patients are commonly regarded as the first victims, healthcare professionals involved in such incidents may also experience significant psychological and physical distress and are referred to as second victims (SVs) [2, 3]. An SV is defined as “any healthcare worker, directly or indirectly involved in an unanticipated adverse patient event, unintentional healthcare error, or patient injury, and who becomes victimized in the sense that they are also negatively impacted” [3]. The range of emotional, physical, and professional responses experienced by SVs—including guilt, anxiety, depression, sleep disturbance, symptoms of post-traumatic stress disorder, and overprotective behavior—is collectively termed the second victim phenomenon (SVP). The term “second victim syndrome” appears in older literature; however, “phenomenon” is now preferred to avoid pathologizing these experiences. Such responses may be associated with time off work or resignation. SVP is estimated to affect 10–58% of healthcare professionals globally [4, 5]. More recent large-scale studies have reported higher prevalence, with over 80% of healthcare professionals experiencing SVP in some settings [6, 7]. These wide variations likely reflect differences in study populations, definitions, and measurement approaches.

In Japan, reported incidences of adverse events and medical errors range from 13% to 73%, compared with approximately 25% in the United States [8–11]. These suggest that many Japanese healthcare professionals are involved in PSIs. The prevalence and persistence of SVP may vary by individual, incident-related, and organizational characteristics, such as punitive culture and the availability of support systems [12, 13]. Although emotional distress, guilt, and self-doubt are core features of SVP reported across healthcare systems, the contexts surrounding incident-reporting culture, punitive measures, and long or shift work hours differ between Japan and other countries. Most critically, research on SVs in Japan is limited, and the development of support systems for SVs remains underdeveloped. Therefore, it is necessary to clarify SVP in Japan and quantify the impact of its organizational culture, support availability, persistence of SVP, turnover intentions, absenteeism, and resilience. Japan’s relatively low racial and cultural diversity (high homogeneity) provides an opportunity to examine psychological responses with less cultural heterogeneity than in more multicultural societies [14].

Furthermore, SV distress affects healthcare professionals who are directly involved in PSIs, as well as those who are indirectly involved as observers [3]; however, little is known about differences in distress, recovery, and support needs between these groups. Their experiences and desired support strategies may differ depending on their roles, and tailored support may be needed for each group.

**Fig. 1:**
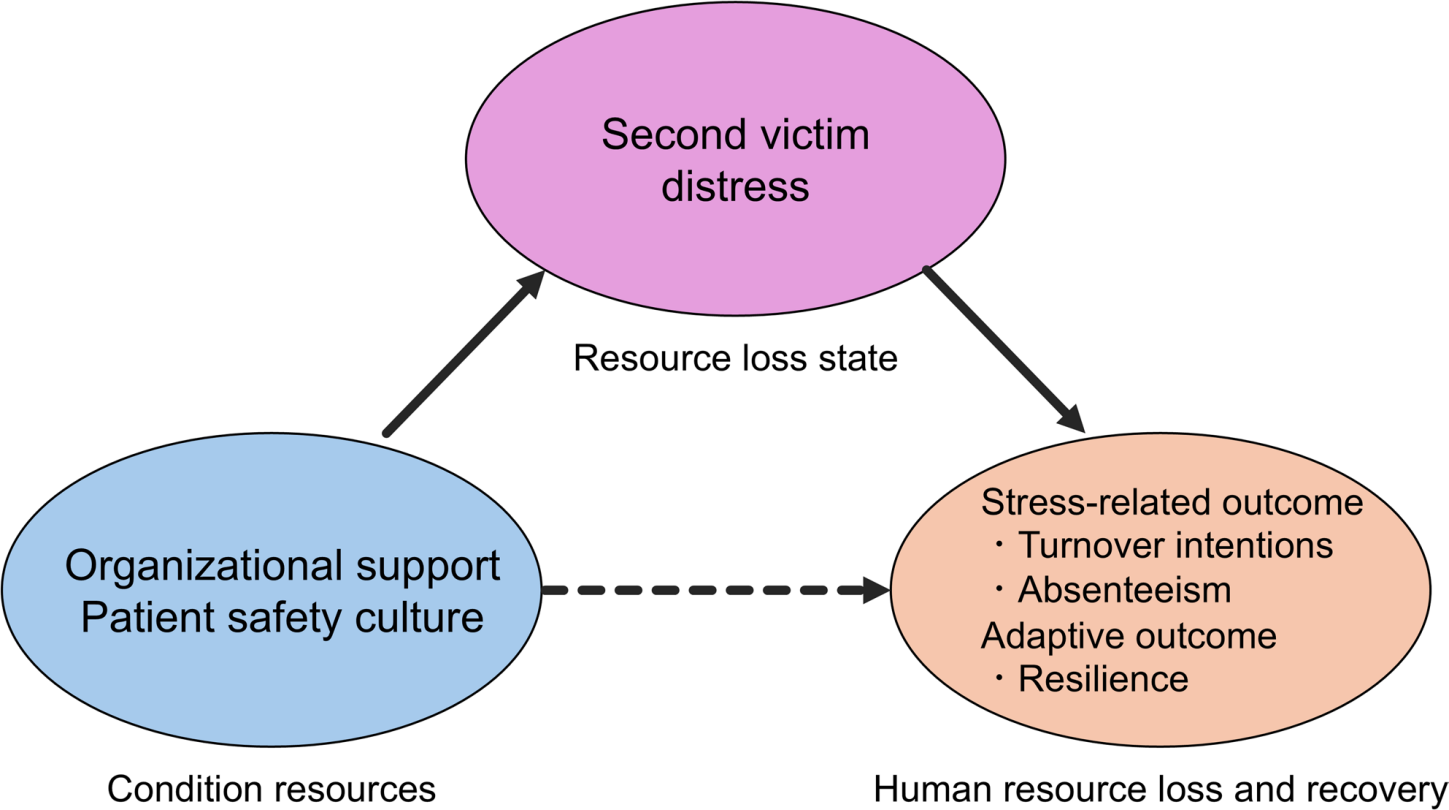
Conceptual framework based on Conservation of Resources Theory. Organizational resources, including organizational support and a nonpunitive patient safety culture, function as condition resources that mitigate resource loss following patient safety incidents. SV distress represents a resource loss state and mediates the relationship between organizational factors and stress-related employment outcomes and adaptive outcomes

This study draws upon stress theories and resource-based frameworks to explain how organizational factors shape outcomes following a PSI. According to the Transactional Model of Stress and Coping, the PSI experience is cognitively appraised and manifests as SV distress, which subsequently triggers stress responses and behavioral outcomes [15]. In parallel, Conservation of Resources Theory posits that resource loss is associated with strong stress responses, whereas resource gain is associated with reduced stress [16]. Organizational support and a nonpunitive patient safety culture function as protective resources that may protect against resource loss. These resources are primarily associated with outcomes through lower SV distress, which represents a state of resource loss. Consistent with this framework, organizational resources are associated with human resource loss and recovery outcomes, including stress-related outcomes such as turnover intentions and absenteeism, as well as the adaptive outcome of resilience. Based on this theoretical framework, we hypothesized that SV distress, conceptualized as a resource loss state, mediates the relationship between organizational factors and employment-related and adaptive outcomes (Fig. 1).

The purpose of this study was to examine the prevalence, impact, and recovery patterns of SVP among healthcare professionals in Japan and to clarify its association with professional outcomes. Specifically, we aimed to (1) quantify the frequency and types of SVP following patient safety incidents (PSIs), (2) identify differences between directly involved staff and observers, and (3) evaluate how organizational support and patient safety culture influence turnover intentions, absenteeism, and resilience. To verify this, we conducted a nationwide, web-based survey called the “JaSeVic study” and applied structural equation modeling and mediation analysis.

## Methods

### Study design and sampling

The study used a web-based, cross-sectional electronic questionnaire survey design. The target population included individuals with experience as medical professionals, with no occupational restrictions. Exclusion criteria were under 18 years of age and an inability to understand Japanese.

Research referral emails with a link to an e-questionnaire were sent to subscribers of the mailing lists of the Japanese Society for Quality and Safety in Healthcare, the Japan Society of Clinical Safety, the Patient Safety Conference of a university hospital, the Japanese Society of Intensive Care Medicine, the Nursing Committee of the Japanese Society of Education for Physicians and Trainees in Intensive Care, and the Japanese Red Cross Society Pharmaceutical Association. The email was sent in September 2024, and the response period was six weeks, ending in mid-October 2024. Reminder emails were sent every two weeks.

### Measurements

The questionnaire incorporated both newly developed items and established instruments from prior studies. It assessed participant demographics, features of their most memorable PSI, awareness of the “second victim” concept and SV support, types of SVP experienced, SVP recovery time, and impact of SVP (Appendix 1).

Participants were allowed to select multiple SVP types, which were derived from manifestations identified in our previous systematic review and meta-analysis [5], synthesizing the literature on second victim experiences. This approach aimed to ensure content validity and comprehensive inclusion of commonly reported SVP symptoms. The recovery time was also developed based on this systematic review [5]. Other items were generated based on prior research and clinical expertise [17]. A panel of experts (SV researchers, patient safety researchers, nurses, and physicians) reviewed the items for relevance and comprehensiveness. A pilot test was then conducted to confirm clarity within the Japanese context. During this process, the content and wording of the items were revised.

Previously validated scales used in the survey included the Agency for Healthcare Research and Quality Hospital Survey on Patient Safety Culture (AHRQ HSOPSC) [18–20] and the Japanese version of the Second Victim Experience and Support Tool-Revised (J-SVESTR) [21, 22]. To reduce order-effect bias, HSOPSC and J-SVESTR items were randomized for each participant [23].

The study examined PSI characteristics, including incident type [24], presence of human error or harm, and severity of harm. Severity was classified using the Medication Error Index developed by the National Coordinating Council for Medication Error Reporting and Prevention [25]. This index is categorized into levels E through I. Level E refers to temporary harm requiring intervention; level F refers to temporary harm requiring initial or prolonged hospitalization; level G refers to permanent harm; level H refers to required intervention necessary to sustain life; and level I refers to a patient’s death.

The HSOPSC is a measure of patient safety culture. We used its subscales “teamwork within units” (four items) and “response to error” (three items). Responses were evaluated on a 5-point Likert scale ranging from 1 (“Strongly disagree”) to 5 (“Strongly agree”), with higher scores indicating better teamwork within units and a more established nonpunitive culture toward errors [18]. Scores for “response to error” were reversed because the items were negatively worded. The validity and reliability of the Japanese version of the HSOPSC have been verified (*χ*^2^ = 11,035, *df* = 753, CFI = 0.89, TLI = 0.88, RMSEA = 0.046, SRMR = 0.044) [19]. The Cronbach’s alpha for the subscales was 0.83 for “teamwork” and 0.71 for “response to error.”

The J-SVESTR consists of two sections: Parts A and B [21, 22]. Part A includes 35 items across seven psychosocial factors (psychological distress, physical distress, colleague support, supervisor support, institutional support, professional self-efficacy, and resilience) and two employment-related factors (turnover intentions and absenteeism). Responses are evaluated using a 5-point Likert scale ranging from 1 (“Strongly disagree”) to 5 (“Strongly agree”). Higher scores indicate stronger SV reactions, a stronger perception of insufficient support, and poor employment outcomes. Percentages were calculated for each factor to indicate the extent to which respondents agreed with the statements, with a factor mean ≥ 4.0 indicating agreement. Part B evaluated seven types of desirable support. These items are rated on a 5-point Likert scale ranging from 1 (“Strongly disagree”) to 5 (“Strongly agree”). A score of 4 or 5 on each of the seven items indicates that support is desired, while a score of 1 or 2 indicates that it is not. Percentages are calculated to indicate the extent to which each form of support is desired. A previous study developed a Japanese version and confirmed its validity and reliability [22]. The construct validity of J-SVESTR (*χ*^2^/*df* = 1.811, RMSEA = 0.060, CFI = 0.871, TLI = 0.854, SRMR = 0.077), internal consistency (Cronbach’s α = 0.68–0.85), and test-retest reliability (ICC = 0.63–0.87) were all acceptable.

### Statistical analysis

Quantitative variables were summarized using mseans (standard deviation [SD]), and qualitative variables were summarized using counts and percentages. Participants were classified into two groups based on whether they were involved in a PSI or were PSI observers, and between-group comparisons were conducted for SVP type, recovery time, impact, and J-SVESTR scores. For between-group comparisons, Student’s t-test was used for quantitative variables, and the chi-square test was used for qualitative variables.

To examine associations between variables and to assess potential multicollinearity, correlation analyses were conducted using Spearman’s correlation coefficient. If the correlation coefficient did not exceed 0.80, multicollinearity was considered absent [26]. Furthermore, in multiple regression analysis, models were adopted where the variance inflation factor for all variables was less than 3.0 [27]. As the primary analysis, structural equation modeling (SEM) was used to verify the research hypothesis. As a sensitivity analysis, the same model was applied to the involvement group, excluding the observation group. Model fit was evaluated using the following criteria, which represent commonly accepted standards for good fit: *χ*^2^/*df* < 3 or 5, CFI > 0.90, TLI > 0.90, RMSEA < 0.05 (good) or 0.05–0.08 (acceptable), SRMR < 0.08 [27, 28].

Mediation analyses were conducted based on the results of the SEM to assess the indirect effects of organizational support and a patient safety culture on the outcomes of SV distress—specifically, turnover intentions, absenteeism, and resilience. Standardized estimates of both indirect and total effects were calculated. A bias-corrected percentile bootstrap method with 5,000 resamples was employed to generate 95% confidence intervals (CIs) for the indirect effects.

We assumed that SV distress after a PSI greatly affects outcomes and conducted a multiple regression analysis to identify factors associated with SV distress. Because 95% CI for standardized coefficients are not typically calculated in multiple regression analysis, unstandardized coefficients *B* (95% CI) and standardized coefficients *β* were reported.

In SEM, mediation analysis, and multiple regression analysis, the scores of the variables for lack of support and resilience were reversed for an easier interpretation of the results. That is, in these analyses, higher scores on organizational support and patient safety culture variables reflect a perception of stronger support and a more positive safety culture, respectively. In this study, “SV distress” was defined as distress resulting from a PSI and was operationalized using three subscales of the J-SVESTR: psychological distress, physical distress, and lower professional self-efficacy. Lower professional self-efficacy is a core response of SVP (loss of professional confidence), and was conceptualized as an integrated aspect of distress alongside psychological and physical reactions. “Organizational support” was defined as support resources recognized after PSI and was integrated into the three subscales of J-SVESTR (colleague support, supervisor support, and institutional support). Patient safety culture was defined as the normative climate within a department and was measured using two subscales of the HSOPSC: teamwork within units and a non-punitive response to errors. Turnover intention, absenteeism, and resilience were selected as organizational key performance indicators to represent how SV distress—as a state of perceived resource depletion—relates to human resource loss and recovery, informed by Conservation of Resources Theory, SVEST-R, and prior research on patient safety culture [16, 29, 30].

An a priori sample size calculator was used to estimate the sample size required for SEM [31, 32]. Under the conditions of two latent variables, 13 observed variables, an effect size of 0.3 (medium), a significance level of 0.05, and statistical power of 0.80, the recommended minimum sample size was 288.

Missing data in the HSOPSC and J-SVESTR Part A were excluded because they were not analyzable. Missing data in J-SVESTR Part B were included in the main analysis but excluded from the descriptive statistics for Part B.

SPSS was used for descriptive statistics, group comparisons, and multiple regression analysis. All the analyses were performed using IBM SPSS Statistics Version 29 and IBM SPSS Amos Version 29 (IBM Corp., Armonk, NY, USA), with a significance level set at *p* < 0.05.

The primary objective of this study was to describe SVP among Japanese healthcare professionals using a multiprofessional integrated sample; therefore, no subgroup analyses by professional category were conducted. Professional categories were recorded solely for descriptive purposes. Profession was recorded for descriptive purposes only. Reporting adhered to the STROBE guideline for cross-sectional studies [33].

## Results

### Characteristics of the respondents and patient safety incidents

The mailing lists included a total of 18,266 registered members. Overall, there were 1,400 responses (7.7%); after excluding 516 responses with incomplete HSOPSC or J-SVESTR data, 884 responses (4.8%) remained for analysis. The responses for each mailing list are shown in Appendix 2. Among the 884 participants who took part in the survey, 486 (55.0%) were female (Table 1). Fewer than half of the participants (*n* = 388, 43.9%) were familiar with the term “second victim.” Only 15.7% reported that their organization had a second victim support system; 62.9% reported its absence, and 20.9% were unsure about the availability of such support. Regarding PSIs, 634 (71.9%) participants reported having been involved in a PSI, while 250 (28.1%) reported having observed one.

**Table 1:** Characteristic data of respondents

Variables	Respondents (*n* = 884)
Female, *n* (%)	486 (55.0)
Age, mean (SD)	50.1 (10.0)
Years of experience as a healthcare professional, mean (SD)	26.1 (10.1)
Occupation, *n* (%)	
Nurse	412 (46.6)
Physician	303 (34.3)
Pharmacist	57 (6.4)
Clinical engineer	33 (3.7)
Therapist	15 (1.7)
Radiation technician	12 (1.4)
Laboratory technician	9 (1.0)
Midwife	10 (1.1)
Dentist	6 (0.7)
Others	27 (3.1)
Knowing the term “second victim”	388 (43.9)
The organization has support for a second victim, *n* (%)	
Yes	139 (15.7)
No	556 (62.9)
Unknown	185 (20.9)
Encounter with patient safety incident, *n* (%)	
Involvement	634 (71.9)
Observation	250 (28.1)
Experience years at the time of patient safety incident, *n* (%)	
< 5	233 (26.4)
6–10	141 (16.0)
11–15	142 (16.1)
> 15	368 (41.6)

Abbreviation: SD = standard deviation

Appendix 3 shows the departments in which PSIs occurred, and Table 2 shows the characteristics of the PSIs. The most frequently reported PSI categories were incidents related to patient care (*n* = 208, 23.5%), medication (*n* = 199, 22.5%), surgery (*n* = 191, 21.6%), and procedure (*n* = 175, 19.8%).

**Table 2:** Characteristics of PSIs

Variables	*n* = 884 (%)
Type of patient safety incident	
Diagnosis-related	81 (9.2)
Medication-related	199 (22.5)
Patient care-related	208 (23.5)
Procedure-related	175 (19.8)
Surgery-related	191 (21.6)
Infection-related	6 (0.7)
Patient suicide	10 (1.1)
Sudden worse	9 (1.0)
Other	5 (0.6)
Error and harm of patient safety incident	
Error, prevented (near-miss)	11 (1.2)
Error, no harm (error)	164 (18.6)
Error, harm (error/adverse event)	464 (52.5)
No error, harm (adverse event)	245 (27.7)
Severity of harm caused by patient safety incident^a^	
No harm	149 (21.9)
E: temporary harm, required intervention	125 (14.1)
F: temporary harm, required initial or prolonged hospitalization	117 (13.2)
G: permanent patient harm	105 (11.9)
H: required intervention necessary to sustain life	86 (9.7)
I: patient’s death	302 (34.2)

^a^ The severity was classified based on the Medication Error Index adopted by the National Coordinating Council for Reporting and Prevention of Medication Errors

Abbreviation: PSI = patient safety incident

### Type of second victim phenomenon

Most respondents (78.1%) reported some form of cognitive and emotional response and/or psychological, physical, or behavioral impact following a PSI (Table 3). Specifically, 689 (77.9%) reported cognitive and emotional responses, 665 (75.2%) reported psychological distress, 540 (61.1%) reported physical distress, and 551 (62.3%) reported behavioral impact. When comparing those directly involved in a PSI with observers, the prevalence of any SVP type was significantly higher among those involved (80.0% vs. 73.2%, *p* = 0.017).

**Table 3:** SVP caused by PSIs

Variables	Overall (*n* = 884)	Involvements (*n* = 634)	Observers (*n* = 250)	*p* value
**Second victim phenomenon**	690 (78.1)	507 (80.0)	183 (73.2)	0.017
*Cognitive and emotional responses*	689 (77.9)	506 (79.8)	183 (73.2)	0.002
Guilt	433 (49.0)	332 (52.4)	101 (40.4)	< 0.001
Loss of self-confidence	248 (40.6)	287 (45.3)	72 (28.8)	< 0.001
Shame	141 (16.0)	112 (17.7)	29 (11.6)	0.008
Worry	339 (38.3)	248 (39.1)	91 (36.4)	0.588
Regret	353 (39.9)	269 (42.4)	84 (33.6)	< 0.001
Despair	213 (24.1)	169 (26.7)	44 (17.6)	0.008
Fear of judgment by colleagues	187 (21.2)	148 (23.3)	39 (15.6)	0.015
Fear of losing the job	82 (9.3)	62 (9.8)	20 (8.0)	0.559
Fear of litigation	235 (26.6)	182 (28.7)	53 (21.2)	0.034
Anger at self	226 (25.6)	176 (27.8)	50 (20.0)	0.012
Anger at others	144 (16.3)	109 (17.2)	35 (14.0)	0.492
Thoughts of hurting oneself	59 (6.7)	41 (6.5)	18 (7.2)	0.694
Desire to work through the incident for a deeper understanding	137 (15.5)	104 (16.4)	33 (13.2)	0.516
Desire to get support from others	128 (14.5)	92 (14.5)	36 (14.4)	0.864
Consider career change	126 (14.3)	99 (15.6)	27 (10.8)	0.039
Other (free comments: sense of responsibility as a manager, empathy for those involved, feelings of helplessness toward those involved, bewilderment, desire to escape, loneliness, fear, distrust, etc.)	34 (3.8)	23 (3.6)	11 (4.4)	
*Psychological distress*	665 (75.2)	489 (77.1)	176 (70.4)	0.001
Anxiety	480 (54.3)	366 (57.7)	114 (45.6)	< 0.001
Depression	234 (26.5)	177 (27.9)	57 (22.8)	0.162
Lethargy	147 (16.6)	106 (16.7)	41 (16.4)	0.735
Recall of the situation at the workplace	424 (48.0)	325 (51.3)	99 (39.6)	0.001
Recall of the situation outside the workplace	395 (44.7)	293 (46.2)	102 (40.8)	0.216
Other (free comments: tension)	3 (0.3)	2 (0.3)	1 (0.4)	
*Physical distress*	540 (61.1)	397 (62.6)	143 (57.2)	0.159
Loss of sleep	253 (28.6)	188 (29.7)	65 (26.0)	0.241
Headaches	79 (8.9)	55 (8.7)	24 (9.6)	0.664
Back pain	20 (2.3)	14 (2.2)	6 (2.4)	0.863
Fatigue	420 (47.5)	310 (48.9)	110 (44.0)	0.231
Loss of appetite	142 (16.1)	105 (16.6)	37 (14.8)	0.485
Nausea	52 (5.9)	41 (6.5)	11 (4.4)	0.240
Nightmare	103 (11.7)	81 (12.8)	22 (8.8)	0.122
Other (free comments: Hand tremors, palpitations, stomachache, tearfulness, increased appetite, shortness of breath, drowsiness, diarrhea, stiff shoulders, etc.)	29 (3.3)	25 (3.9)	4 (1.6)	
*Impact on behavior*	551 (62.3)	399 (62.9)	152 (60.8)	0.817
Lack of concentration	344 (38.9)	250 (39.4)	97 (37.6)	0.556
Defensive, overprotective behavior	229 (25.9)	167 (26.3)	62 (24.8)	0.769
Aggressive, risky behavior	44 (5.0)	32 (5.0)	12 (4.8)	0.879
Use of alcohol or other substances	80 (9.0)	54 (8.5)	26 (10.4)	0.423
Loss of interest in daily activities	150 (17.0)	115 (18.1)	35 (14.0)	0.140
Other (free comments: behavior related to leaving work, dressing as usual, being on edge, immersing oneself in other things, taking positive steps toward improvement, becoming nervous)	22 (2.5)	12 (1.9)	10 (4.0)	

Abbreviation: PSI = patient safety incident; SVP = second victim phenomenon

Regarding cognitive and emotional responses, individuals in the involvement group reported significantly higher rates of guilt (52.4% vs. 40.4%, *p* < 0.001), loss of self-confidence (45.3% vs. 28.8%, *p* < 0.001), shame (17.7% vs. 11.6%, *p* = 0.008), regret (42.4% vs. 33.6%, *p* < 0.001), despair (26.7% vs. 17.6%, *p* = 0.008), and fear of judgment by colleagues (23.3% vs. 15.6%, *p* = 0.015).

Psychological distress was also significantly more prevalent in the involvement group than among observers (77.1% vs. 70.4%, *p* = 0.001), particularly symptoms of anxiety (57.7% vs. 45.6%, *p* < 0.001), and intrusive recall (51.3% vs. 39.6%, *p* < 0.001). No significant differences were observed between the two groups for physical distress or behavioral impact.

### Recovery time and impact of second victim phenomenon

Among the respondents, 194 (21.9%) reported no symptoms, and 180 (20.4%) recovered within one month. Recovery within one year was reported by 175 respondents (19.8%), whereas 123 (13.9%) required more than one year to recover, and 86 (9.7%) continued to report symptoms (Table 4). There were no significant differences between those involved in a PSI and observers in terms of recovery time (*p* = 0.400). Furthermore, a positive correlation was observed between the severity of PSI damage and recovery time (*r*_*s*_ = 0.151, *p* < 0.001).

**Table 4:** Recovery time and impact of SVP

Variables	Overall (*n* = 884)	Involvements (*n* = 634)	Observations (*n* = 250)	*p* value
*Recovery time*				0.400
No symptoms	194 (21.9)	127 (20.0)	67 (26.8)	
≤ 1 day	13 (1.5)	11 (1.7)	2 (0.8)	
≤ 1 week	113 (12.8)	81 (12.8)	32 (12.8)	
≤ 1 months	180 (20.4)	133 (21.0)	47 (18.8)	
≤ 1 year	175 (19.8)	127 (20.0)	48 (19.2)	
> 1 year	123 (13.9)	93 (14.7)	30 (12.0)	
> 1 year, symptoms remain	86 (9.7)	62 (9.8)	24 (9.6)	
*Impact on work*				0.390
Overcame and used the experience to improve	505 (57.1)	371 (58.5)	134 (53.6)	
Continued to work, no symptoms	272 (30.8)	185 (29.2)	87 (34.8)	
Continued to work, but with symptoms	69 (7.8)	49 (7.7)	20 (8)	
Left work	38 (4.3)	29 (4.6)	9 (3.6)	

Abbreviation: SVP = second victim phenomenon

Most respondents (*n* = 505, 57.1%) reported overcoming the event and using the experience for improvement; 7.8% continued working with symptoms, and 4.3% left their position. Work-related impact did not differ significantly between the involvement and observer groups (*p* = 0.390).

### The experience of the second victim

The results of the descriptive analysis of the J-SVESTR Part A showed that the overall score for SV distress was 2.89 (0.87) (Table 5). The subscale scores were: psychological distress 3.26 (0.98), physical distress 2.62 (1.02), and lower professional self-efficacy 2.84 (0.94). The overall score for lack of organizational support was 2.78 (0.74). The subscale scores were: colleague support 2.42 (0.81), supervisor support 2.75 (0.97), and institutional support 3.31 (0.91). Outcome subscale mean scores were: turnover intention 2.37 (SD = 1.04), absenteeism 1.60 (SD = 0.83), and lower resilience 2.18 (SD = 0.72).

**Table 5:** Descriptive statistics and group comparisons of J-SVESTR Part A

Variables	Overall (*n* = 884)		Involvements (*n* = 634)		Observations (*n* = 250)		*p* value
	Mean (SD)	Agreement (%)	Mean (SD)	Agreement (%)	Mean (SD)	Agreement (%)	
*Second victim distress*	2.89 (0.87)	105 (11.9)	2.93 (0.90)	82 (12.9)	2.77 (0.85)	23 (9.2)	0.013
Psychological distress	3.26 (0.98)	256 (29.0)	3.32 (0.97)	193 (30.4)	3.13 (0.99)	63 (25.2)	0.012
Physical distress	2.62 (1.02)	107 (12.1)	2.65 (1.03)	86 (13.6)	2.53 (0.98)	21 (8.4)	0.101
Lower professional self-efficacy	2.84 (0.94)	140 (15.8)	2.90 (0.95)	109 (17.2)	2.71 (0.91)	31 (12.4)	0.008
*Lack of organizational support*	2.78 (0.74)	60 (6.8)	2.80 (0.77)	53 (8.4)	2.72 (0.65)	7 (2.8)	0.127
Colleague support	2.42 (0.81)	44 (5.0)	2.44 (0.84)	38 (6.0)	2.36 (0.71)	6 (2.4)	0.179
Supervisor support	2.75 (0.97)	117 (13.2)	2.76 (1.00)	91 (14.4)	2.74 (0.88)	26 (10.4)	0.745
Institutional support	3.31 (0.91)	263 (29.8)	3.36 (0.93)	203 (32.0)	3.19 (0.69)	60 (24.0)	0.010
*Outcomes*							
Turnover intentions	2.37 (1.04)	90 (10.2)	2.37 (1.05)	69 (10.9)	2.35 (1.00)	21 (8.4)	0.722
Absenteeism	1.60 (0.83)	17 (1.9)	1.58 (0.81)	18 (2.8)	1.65 (0.88)	10 (4.0)	0.299
Lower Resilience	2.18 (0.72)	22 (2.5)	2.18 (0.73)	17 (2.7)	2.18 (0.69)	5 (2.0)	0.916

Abbreviation: SD = standard deviation; SVEST-R = Second Victim Experience Support Tool-Revised

According to the SVEST-R scoring guidelines [21], 256 respondents (29%) met the threshold for psychological distress, 107 (12.1%) met the threshold for physical distress, and 140 (15.8%) met the threshold for lower self-efficacy. In terms of organizational support subscales, 44 respondents (5.0%) perceived a lack of colleague support, 117 (13.2%) perceived a lack of supervisor support, and 263 (29.8%) perceived a lack of institutional support. In addition, 90 respondents (10.2%) met the threshold for turnover intention, 17 (1.9%) reported absenteeism, and 22 (2.5%) met the threshold for lower resilience.

The involvement group showed significantly higher scores for SV distress (2.93 vs. 2.77, *p* = 0.013). Specifically, psychological distress (3.32 vs. 3.13, *p* = 0.012) and lower professional self-efficacy (2.90 vs. 2.71, *p* = 0.008) were significantly higher in the involved group. Additionally, the involvement group perceived a greater lack of institutional support than the observer group (3.36 vs. 3.19, *p* = 0.010). No significant group differences were observed for outcome variables, including turnover intention (*p* = 0.722), absenteeism (*p* = 0.299), and lower resilience (*p* = 0.916).

### The relationship between organizational support and patient safety culture, second victim distress, and outcomes

**Fig. 2:**
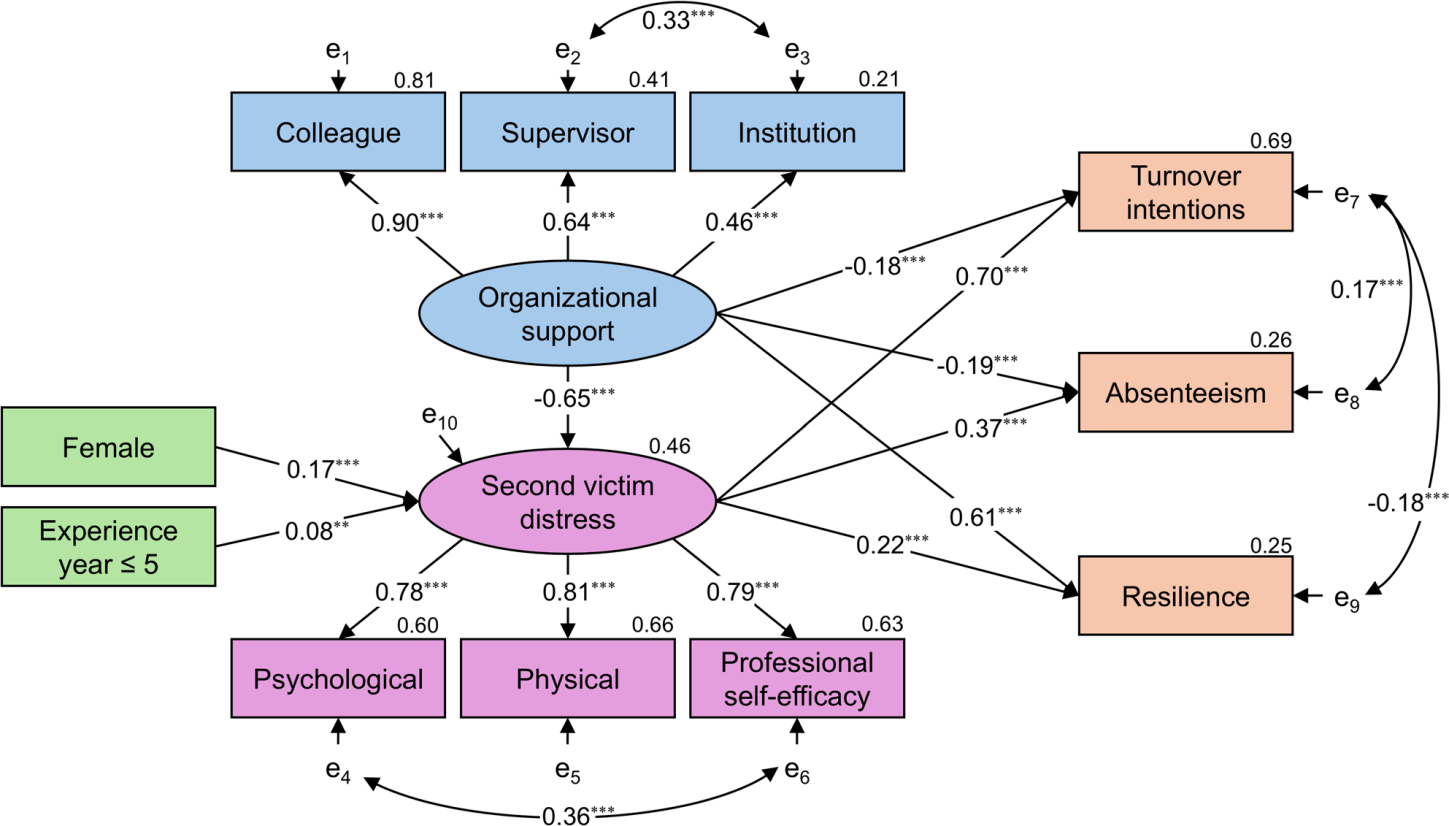
SEM showing the associations between organizational support, SV distress, and outcomes Standardized estimates are shown (*p < 0.05; **p < 0.01; ***p < 0.001). Latent variables are indicated by ellipses, observed variables by rectangles, and e₁–e₁₀ are error terms. The coefficient of determination *R*^2^ is shown in the upper right of each endogenous variable

As shown in Appendix 4, no excessively high correlations were observed among variables, supporting their suitability for SEM analysis. SEM 1 illustrates the associations between organizational support, SV distress, and outcomes (Fig. 2). Model 1 goodness-of-fit was as follows: *χ*^2^ = 210.817, *p* < 0.001, *χ*^2^/*df* = 5.856, CFI = 0.953, TLI = 0.928, RMSEA = 0.074, and SRMR = 0.041. Although *χ*^2^/*df* exceeds 5, considering the sample size and model complexity, the overall goodness-of-fit based on multiple metrics was judged acceptable. Organizational support was significantly associated with SV distress (*β* = −0.65, *p* < 0.001), turnover intention (*β* = −0.18, *p* < 0.001), absenteeism (*β* = −0.19, *p* < 0.001), and resilience (*β* = 0.61, *p* < 0.001). SV distress was significantly associated with turnover intention (*β* = 0.70, *p* < 0.001), absenteeism (*β* = 0.37, *p* < 0.001), and resilience (*β* = 0.22, *p* < 0.001).

**Fig. 3:**
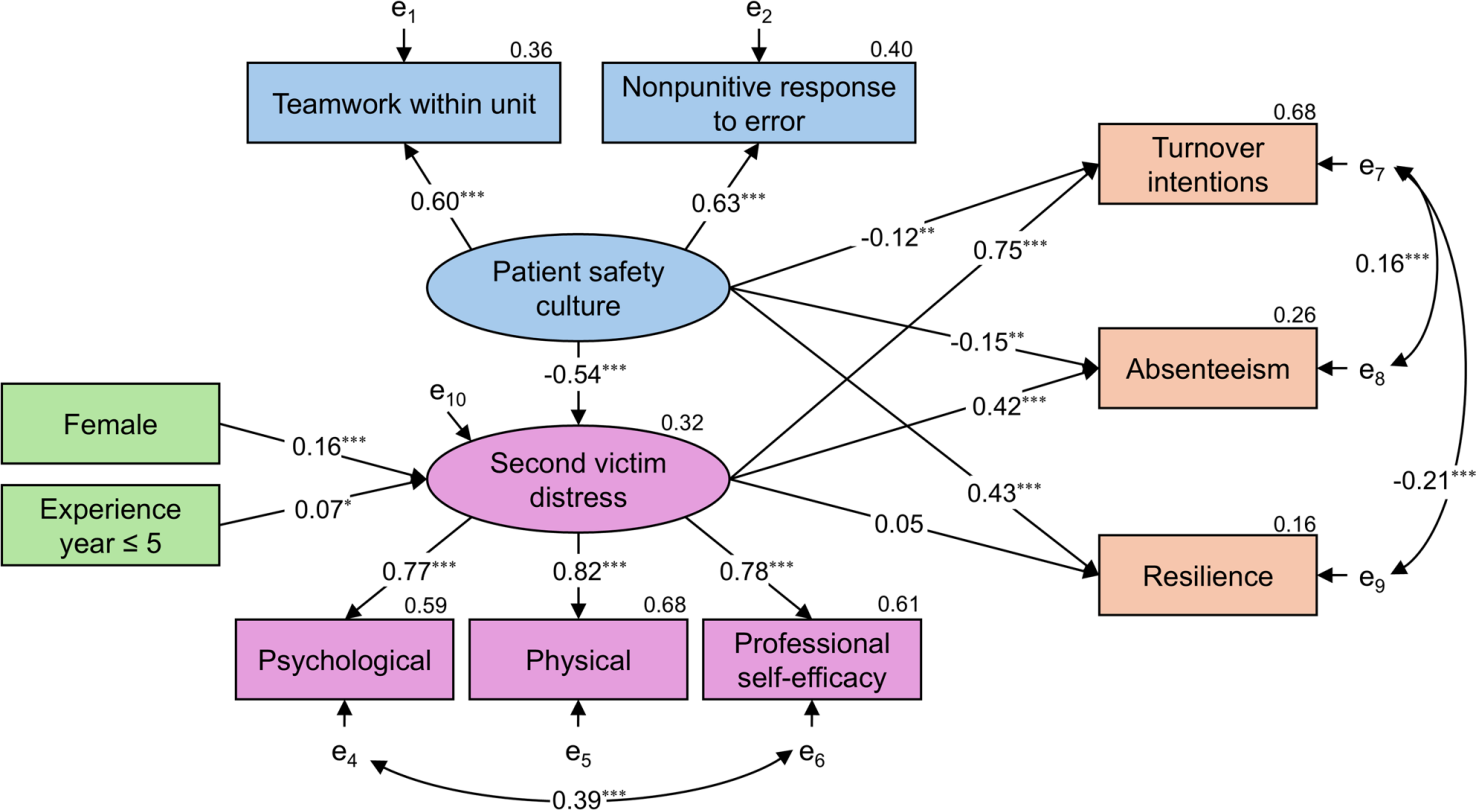
SEM showing the associations between patient safety culture, SV distress, and outcomes Standardized estimates are shown (*p < 0.05; **p < 0.01; ***p < 0.001). Latent variables are indicated by ellipses, observed variables by rectangles, and e₁–e₁₀ are error terms. The coefficient of determination R^2^ is in the upper right of each endogenous variable

SEM 2 shows the relationship between a patient safety culture and SV distress on outcomes (Fig. 3). Model 2 goodness-of-fit was as follows: *χ*^2^ = 144.352, *p* < 0.001, *χ*^2^/*df* = 5.155, CFI = 0.959, TLI = 0.934, RMSEA = 0.069, and SRMR = 0.043. Although *χ*^2^/*df* exceeds 5 in this model as well, we judged the overall goodness of fit to be acceptable, similar to Model 1. A positive patient safety culture was significantly associated with SV distress (*β* = −0.539, *p* < 0.001), turnover intention (*β* = −0.123, *p* = 0.003), absenteeism (*β* = −0.152, *p* = 0.003), and resilience (*β* = 0.427, *p* < 0.001). SV distress was significantly associated with turnover intention (*β* = 0.751, *p* < 0.001) and absenteeism (*β* = 0.416, *p* < 0.001). SV distress was not significantly associated with resilience (*β* = 0.427, *p* = 0.355).

A sensitivity analysis was conducted for both SEM 1 and SEM 2 using only the involvement group, excluding the observer group. The results were consistent with those of the primary analyses for both models (Appendix 5 and 6). The goodness-of-fit for these models was acceptable.

Mediation analyses examined the indirect and total effects of organizational support and a positive patient safety culture on outcomes in SV distress (Table 6). In model 1, organizational support showed significant indirect associations with lower turnover intention (indirect effect = −0.457, 95% CI [−0.532, −0.395], *p* < 0.001), absenteeism (−0.239, 95% CI [−0.310, −0.169], *p* < 0.001), and resilience (−0.141, 95% CI [−0.248, −0.054], *p* = 0.001). The corresponding total effects were −0.632 for turnover intention, −0.429 for absenteeism, and 0.471 for resilience. In model 2, a positive patient safety culture showed significant indirect associations with lower turnover intention (−0.405, 95% CI [−0.510, −0.309], *p* < 0.001) and lower absenteeism (−0.224, 95% CI [−0.293, −0.168], *p* < 0.001), but not with resilience (−0.026, 95% CI [−0.163, 0.042], *p* = 0.497). The total effects were −0.528 for turnover intention, −0.377 for absenteeism, and 0.400 for resilience.

**Table 6:** Indirect and total effects of support and patient safety culture on outcomes

Outcomes	Indirect effect (*β*)	95% confidence interval		*p* value	Total effect
		Lower	Upper		
**Model 1: Organizational support →** ** Second victim distress → Outcomes**					
Turnover intentions	-0.457	-0.532	-0.395	< 0.001	-0.632
Absenteeism	-0.239	-0.310	-0.169	< 0.001	-0.429
Resilience	-0.141	-0.248	-0.054	0.001	0.471
**Model 2: Patient safety culture → Second victim distress → Outcomes**					
Turnover intentions	-0.405	-0.510	-0.309	< 0.001	-0.528
Absenteeism	-0.224	-0.293	-0.168	< 0.001	-0.377
Resilience	-0.026	-0.163	0.042	0.497	0.400

The 95% confidence interval for the indirect effect was calculated using bias-corrected percentile bootstrap method (5000 samples)

### Factors related to second victim distress

**Fig. 4:**
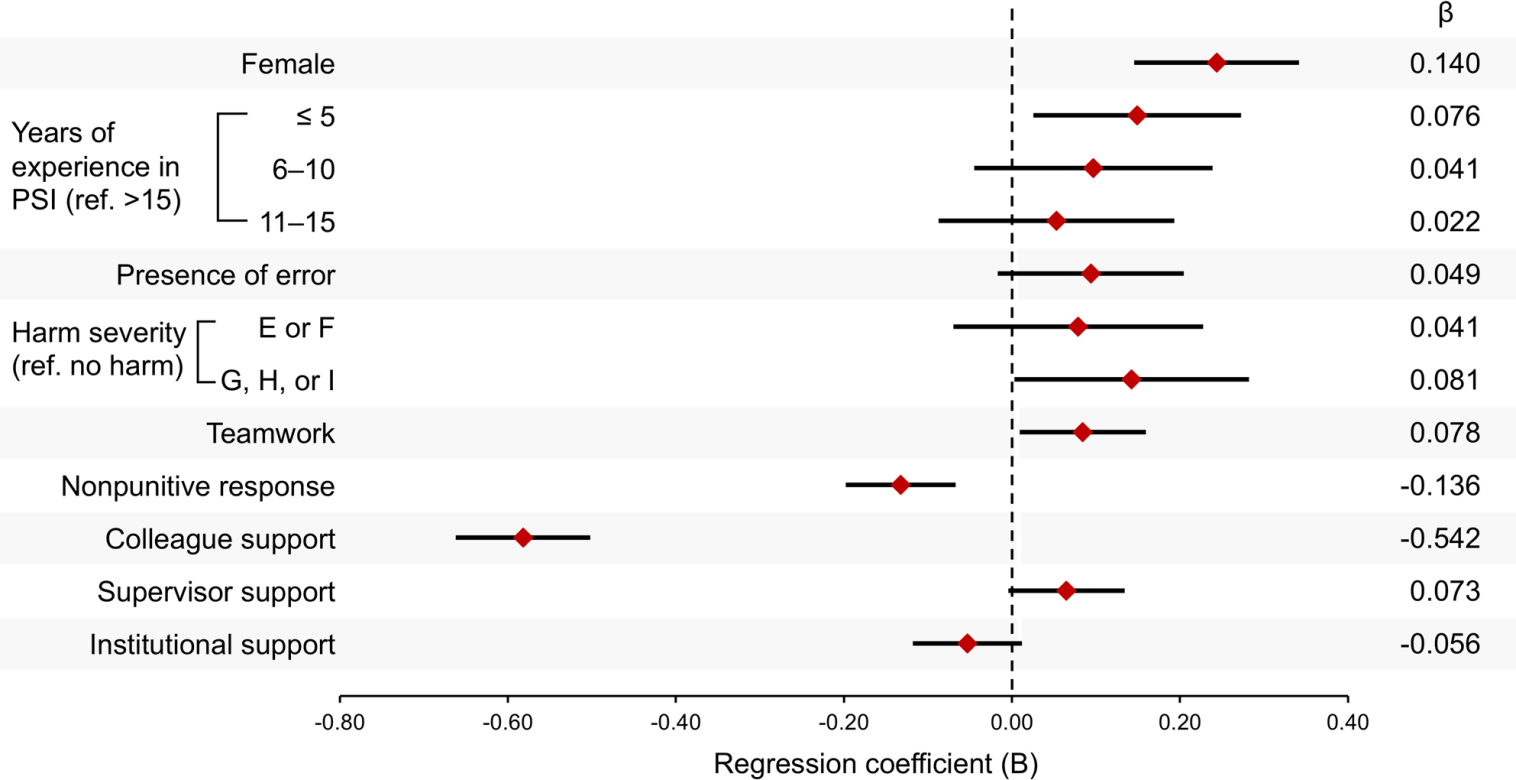
Multiple regression analysis of factors associated with SV distress The forest plot shows the regression coefficients (*B*), 95% confidence intervals, and standardized regression coefficients (*β*) for associated factors of SV distress. Significant protective factors included nonpunitive responses to human error and colleague support. Female gender and less clinical experience were associated with greater distress. Reference groups: >15 years of experience in PSI and no harm severity

Fig. 4 illustrates the regression coefficients for predictors of SV distress. Being female (*B* = 0.244, 95% CI [0.148, 0.339]), having five years or less of professional experience (*B* = 0.149, 95% CI [0.028, 0.270]), and greater harm severity (G, H, or I: *B* = 0.142, 95% CI [0.006, 0.279]) were associated with higher levels of SV distress. Positive patient safety and support factors were also associated with SV distress. Specifically, greater perceived teamwork (*B* = 0.084, 95% CI [0.012, 0.157]) was linked to higher levels of SV distress, whereas a more nonpunitive response (*B* = −0.132, 95% CI [−0.195, −0.070]) and greater colleague support (*B* = −0.582, 95% CI [−0.659, −0.505]) were strongly associated with lower levels of SV distress. Supervisor and institutional support had no significant association.

### Desired support for the second victim

Results from the J-SVESTR Part B indicated that 661 respondents (76.3%) desired “a respected peer to discuss the details of what happened,” 632 (73.0%) desired “a specified peaceful location that is available to recover and recompose after one of these types of events,” and 602 (69.5%) desired “the ability to immediately take time away from my unit for a little while” (Table 7).

**Table 7:** Desired SV support (J-SVESTR Part B)

Desired support option	Overall (*n* = 866)		Involvements (*n* = 623)		Observations (*n* = 243)		*p* value
	Mean (SD)	Agreement (%)	Mean (SD)	Agreement (%)	Mean (SD)	Agreement (%)	
1. The ability to immediately take time away from my unit for a little while	3.79 (0.98)	602 (69.5)	3.78 (0.97)	431 (69.2)	3.81 (1.01)	171 (70.4)	0.676
2. A specified peaceful location that is available to recover and recompose after one of these types of events	3.87 (0.91)	632 (73.0)	3.87 (0.90)	454 (72.9)	3.88 (0.95)	178 (73.3)	0.890
3. A respected peer to discuss the details of what happened	3.97 (0.94)	661 (76.3)	3.99 (0.93)	483 (77.5)	3.92 (0.96)	178 (73.3)	0.347
4. An employee assistance program that can provide free counseling to employees outside of work	3.73 (1.04)	569 (65.7)	3.72 (1.05)	402 (64.5)	3.78 (1.02)	167 (68.7)	0.398
5. A discussion with my manager or supervisor about the incident	3.75 (1.01)	589 (68.0)	3.71 (1.05)	417 (66.9)	3.88 (0.91)	172 (70.8)	0.026
6. The opportunity to schedule a time with a counselor at my hospital to discuss the event	3.72 (1.07)	561 (64.8)	3.73 (1.06)	403 (64.7)	3.69 (1.08)	158 (65.0)	0.597
7. A confidential way to get in touch with someone 24 h a day to discuss how my experience may be affecting me	3.44 (1.07)	450 (52.0)	3.43 (1.05)	320 (51.4)	3.47 (1.11)	130 (53.5)	0.689

Data for 18 individuals was missing and was excluded

Abbreviation: SD = standard deviation; SVEST-R = Second Victim Experience Support Tool-Revised

The comprehensive graphical summary of this study findings, including the prevalence, recovery patterns, impact, and the structural equation model of SVP, are illustrated in Appendix 7.

## Discussion

This study conducted a large-scale, nationwide, web-based survey of 884 healthcare professionals from diverse disciplines. The findings provide a comprehensive overview of the SVP in Japan. Approximately 80% of the respondents had experienced the negative impact of PSIs. More than half viewed their experiences as opportunities for growth, recovery often required extended periods, and 10% reported persistent symptoms beyond one year. Healthcare professionals directly involved in PSIs reported significantly higher levels of SV distress and perceived a greater lack of institutional support compared to observers. Moreover, using the SVEST-R, this study demonstrates that SVP is associated not only with SV distress, turnover intention, and absenteeism but also with resilience. SEM showed that organizational support systems and a positive patient safety culture were both directly and indirectly associated with reduced turnover intention and absenteeism, and with enhanced resilience, primarily via reduced SV distress.

These findings align with prior studies reporting that most healthcare professionals recover from SVP within one month, although approximately 10% continue to experience long-term symptoms [5, 6, 13, 17, 34]. The broader range of psychological and physical responses captured in this study suggests that healthcare professionals may experience a more diverse set of SVP symptoms than those measured by tools like the SVEST alone.

An important contribution of this study is the clear differentiation between healthcare professionals directly involved in PSIs and those who merely observed them. Those directly involved exhibited a broader range of symptoms and reported greater SV distress, emphasizing the need for targeted support. However, the relatively modest difference in overall SV distress scores between the two groups also underscores the importance of providing interventions for both directly and indirectly involved healthcare professionals. This is consistent with the latest SV definition published in the European Researchers’ Network Working on Second Victims’ consensus statement: “any healthcare worker, directly or indirectly involved in” [3]. Furthermore, as a practical program, AHRQ’s CANDOR addresses the need for team-based support [35]. The forYOU program, a pioneering SV support model, integrates individual-level support with team-based debriefings [36, 37]. Notably, compared to studies conducted in the United States, Malaysia, and Italy, Japanese healthcare professionals reported substantially higher perceptions of insufficient institutional support (29.8%) [21, 38, 39]. This highlights a critical gap in Japan’s organizational frameworks for supporting SVs.

Both organizational support and a positive patient safety culture were shown to mitigate SV distress and, consequently, reduce turnover intentions and absenteeism and enhance resilience. Mediation analyses confirmed these indirect pathways. Supportive organizational environments may help alleviate feelings of guilt and isolation, thereby protecting healthcare professionals from long-term psychological strain. Previous studies have found that nonpunitive responses and a positive patient safety culture are associated with reduced SV distress [29, 30]. When SV distress is not adequately addressed, it may contribute to sustained psychological burden, reduced motivation, and heightened turnover intention [40, 41]. This pattern was also observed in the SEM developed for the present study. In addition to indirect effects via SV distress, direct associations between organizational support, safety culture, and outcomes are consistent with Conservation of Resources Theory, which posits that resource gain offers both immediate and mediated benefits [16]. Supportive environments—through strategies such as structured peer support and nonpunitive debriefing—may not only buffer distress but also directly enhance perceived stability and psychological safety, thereby fostering resilience and lowering turnover risk. This dual pathway underscores the importance of comprehensive organizational strategies that both prevent distress and build enduring resource pools.

The multiple regression analysis determined the following to be significantly associated with greater distress: female gender, less than five years of professional experience, harm severity, and better teamwork. The association of female gender and limited professional experience with increased vulnerability to SV distress has also been reported in prior research [42]. Overall, patient safety culture functioned as a condition resource. However, surprisingly, when focusing specifically on teamwork, higher levels of teamwork were associated with greater SV distress. It might partially reflect that cohesive teams are more open to discussing errors and distress, thus increasing both awareness and reporting. Furthermore, considering Japanese culture, this finding might be interpreted as follows. First, according to Hofstede’s Cultural Dimensions Theory, Japan is characterized by an exceptionally high “Masculinity” score [14]. This cultural trait fosters an “errorless imperative” that prioritizes achievement and success while maintaining a low tolerance for failure [43]. The severe psychological reactions to errors identified in this study directly reflect these cultural characteristics. Accordingly, these findings may serve as an important reference point for healthcare organizations in countries with similar cultural value systems. Second, Japan holds an intermediate position on the “Individualism–Collectivism” dimension, resulting in unique team dynamics that differ from those in primarily individualistic or collectivist societies [14]. Our finding regarding the “teamwork paradox”—where positive teamwork can inadvertently exacerbate stress—serves as a valuable model for understanding how SVP manifests in nations with comparable intermediate cultural backgrounds. Further qualitative research is needed to explore this mechanism.

Colleague support emerged as a critical buffer against SV distress, echoing findings from previous studies [44]. A nonpunitive culture not only facilitates error reporting and organizational learning but also alleviates fears of personal blame [45]. These findings suggest that enhancing SV support systems and fostering a positive patient safety culture may contribute to lower SV distress, turnover intention, and absenteeism among healthcare professionals. Notably, support from colleagues appears to have a particularly strong impact on alleviating SV distress [46]. Clinically, peer support programs (e.g., forYOU, RISE) and nonpunitive ‘Just Culture’ reforms are actionable, with emerging consensus indicators to evaluate SV support implementation [37, 47, 48]. The J-SVESTR Part B results prioritize peer support, time away from the unit, and access to a peaceful location for recovery. Based on our findings and the ERNST five-level framework [49]—which includes prevention, self-care, peer support/triage, structured professional support, and clinical support—we propose near-term implementation steps for Japan:

1. Implementing structured peer‑support programs (e.g., institutional policy, 24/7 access, trained peer responders).
2. Establishing psychologically safe, nonpunitive debriefings after PSIs.
3. Embedding SVP training (prevention/self‑care, early detection) into patient safety curricula and staff well‑being strategies.

In Japan, implementing these support strategies for SVs could potentially reduce SVP after PSI, improve employment outcomes, and strengthen resilience.

### Limitation

This study has several limitations. First, due to the recruitment strategy, the sample was non-probabilistic and therefore cannot be assumed to represent the entire Japanese healthcare population. In addition, the response rate was relatively low. Sampling bias cannot be ruled out, as participants were recruited via mailing lists that may have disproportionately attracted individuals with a pre-existing interest in patient safety, potentially inflating estimates of SVP awareness or experience. Consequently, caution is needed in interpreting these results, as they may not generalize to healthcare professionals not affiliated with academic societies or to populations uninterested in patient safety. Future work should consider probability sampling/weighting and targeted recruitment beyond safety networks. That said, as previously noted, Japan has relatively low racial and cultural diversity, which allowed this study to examine “the influence of cultural background on psychological responses” in a less confounded environment compared to multicultural societies [14]. Second, reliance on self-reported recollections of the most memorable PSI introduces recall bias, limiting accurate assessments of immediate psychological effects and institutional support. However, this approach captures deeply influential experiences and, by avoiding predefined case types, allows for a broader view of PSI impacts in clinical settings. Third, social desirability may lead some respondents, particularly in a context where harmony and face-saving are highly valued, to underreport distress, absenteeism, or turnover intentions, or to emphasize “learning and growth” rather than ongoing psychological burden. Furthermore, overconfidence and professional identity—factors highlighted by Bushuven et al.—may cause healthcare professionals to underestimate their vulnerability or their involvement in errors or adverse events [50]. Fourth, the cross-sectional design of this study precludes causal inferences. Future longitudinal and cohort studies are warranted to clarify the temporal dynamics and lasting impacts of SVP. Fifth, this study did not examine occupation-specific differences or conduct subgroup analyses, limiting the ability to assess variation across professional roles. Such analyses are planned for future secondary research, focusing on mechanisms and variability at the occupation level. Finally, because we restricted the index event to PSIs, emotionally distressing events that do not fall under this definition may not have been captured as SVP, potentially leading to an underestimation of SVP compared with studies applying the broader definition. Nonetheless, this study is the first nationwide survey in Japan to comprehensively evaluate SVP using the validated Japanese version of the SVEST-R. By also capturing resilience, it provides a more nuanced understanding of SVP among Japanese healthcare professionals. These insights are expected to raise awareness and guide the development of effective support systems in Japan.

## Conclusions

This nationwide survey is the first to comprehensively investigate SVP among healthcare professionals in Japan using the validated J-SVESTR tool. The findings indicate that diverse SVP experiences occur following PSIs and that most respondents reported having experienced SV distress. Additionally, healthcare professionals directly involved in PSIs were more likely to recognize SVP and perceive a lack of support compared to observers. Importantly, organizational support and a positive patient safety culture were significantly associated with lower SV distress, turnover intention, absenteeism, and higher resilience. These associations were primarily mediated through SV distress.

These results highlight the urgent need to establish structured support systems and foster psychologically safe environments that prioritize nonpunitive approaches and colleague support in Japan. Strengthening such frameworks could contribute not only to protecting the mental health of healthcare professionals but also to improving patient safety and enhancing workforce retention.

## Supplementary Information

Below is the link to the electronic supplementary material.

Supplementary Material 1

## Data Availability

The datasets used and/or analyzed during the current study are available from the corresponding author on reasonable request.

## Abbreviations

AHRQ: Agency of Healthcare Research and Quality
CFI: Comparative fit index
CI: Confidence interval
HSOPSC: Hospital Survey on Patient Safety Culture
ICC: Intraclass correlation coefficient
J-SVESTR: The Japanese version of the Second Victim Experience and Support Tool-Revised
PSI: Patient safety incident
RMSEA: Root mean square error of approximation
SD: Standard deviation
SEM: Structural equation modeling
SV: Second victim
SVP: Second victim phenomenon
SRMR: Standardized root mean squared residual
TLI: Tucker-Lewis index
