# Supplementary material for: The second victim phenomenon in Japan: prevalence, impact, and associated factors— the JaSeVic study
Source: BMC Health Serv Res. 2026 Feb 25;26:441. doi: 10.1186/s12913-026-14251-5 (PMC13040824; doi:10.1186/s12913-026-14251-5)

**Appendix**

**For: The Second Victim Phenomenon in Japan: Prevalence, Impact, and Associated Factors — The JaSeVic Study**

This appendix provides additional materials supporting the main manuscript, including the original survey questionnaire, details of participant recruitment, descriptive information on patient safety incidents, and the results of correlation and structural equation modeling analyses, and a comprehensive summary of the study findings.

**Contents**

Appendix 1. Original items in survey questionnaire

Appendix 2. Mailing list used for recruitment

Appendix 3. Department in which PSIs occurred

Appendix 4. Spearman’s correlation matrix between variables used in the SEM analysis

Appendix 5. SEM for involvement group in PSIs showing the associations between organizational support, SV distress, and outcomes

Appendix 6. SEM for involvement group in PSIs showing the associations between patient safety culture, SV distress, and outcomes

Appendix 7. Comprehensive summary of the JaSeVic study findings.

Appendix 1. Original items in survey questionnaire

The original questionnaire was developed in Japanese. The English version presented here is a direct translation with slight modifications for readability and international comprehensibility.

Section A. Demographic Information

Please tell us about yourself.

Q1. Age (18–70): ______

Q2. Gender: (male/female)

Q3. Professional occupation:

□ Physician / Medical Doctor

□ Dentist

□ Nurse

□ Licensed Practical Nurse (LPN)

□ Nursing Assistant

□ Midwife

□ Physical Therapist

□ Occupational Therapist

□ Speech Therapist

□ Orthoptist

□ Pharmacist

□ Radiological Technologist

□ Medical Technologist

□ Clinical Engineer

□ Dental Hygienist

□ Medical Social Worker

□ Psychologist

□ Public Health Nurse

□ Caregiver

□ Registered Dietitian

□ Medical Clerk

□ Hospital Cook

□ Other (please specify)

Q4. Years of experience as a healthcare professional (0–70): ______

Q5. Are you currently in a managerial position? (Yes/No)

Q6. Before this survey, were you familiar with the term *“second victim”*? (Yes/No)

Q7. Does your facility provide support (peer support, support programs, etc.) for second victims? (Yes/No/I don't know)

Q8. In your professional career, have you ever observed or been directly involved in a patient safety incident*?
(*Patient safety incidents refer to adverse events, medical errors, and near misses. They are not necessarily due to negligence. They include both cases where harm occurred to the patient and those where no harm occurred. They also include both cases where the incident actually occurred and those where it did not.)

□ I have observed

□ I have been directly involved

□ I have never

Section B. About the Most Memorable Patient Safety Incident

Please recall the most memorable patient safety incident you have observed or been involved in.

We will ask about the circumstances at the time of the patient safety incident*.

(*Patient safety incidents refer to adverse events, medical errors, and near misses. They are not necessarily due to negligence. They include both cases where harm occurred to the patient and those where no harm occurred. They also include both cases where the incident actually occurred and those where it did not.)

Q9. Age at the time:

□ ≤ 25

□ 26 – 30

□ 31 – 40

□ 41 – 50

□ 51 – 60

□ 61 – 70

□ > 70

Q10. Years of experience as a healthcare professional at the time:

□ < 1

□ 1 – 5

□ 6 – 10

□ 11 – 15

□ ≥ 16

Q11. Which department did that incident occur in?

□ Medical ward

□ Surgical ward

□ Outpatient department

□ Emergency department (ED)

□ Operating room / Surgical unit

□ Intensive Care Unit

□ High Care Unit

□ Pediatric Intensive Care Unit

□ Neonatal Intensive Care Unit

□ Growing Care Unit

□ Pediatrics

□ Obstetrics and Gynecology

□ Anesthesiology

□ Radiology

□ Rehabilitation

□ Pharmacy

□ Clinic

□ Nursing home

□ Other (please specify)

Section C. About the Impact of the Patient Safety Incident on you

Being involved in an incident related to patient safety can cause psychological and physical symptoms.

What symptoms did you experience after the incident?

Q12. How did you think about it? (Multiple selections possible)

□ Guilt

□ Loss of self-confidence

□ Shame

□ Worry

□ Regret

□ Despair

□ Fear of judgment by colleagues

□ Fear of losing the job

□ Fear of litigation

□ Anger at self

□ Anger at others

□ Thoughts of hurting oneself

□ Desire to work through the incident for a deeper understanding

□ Desire to get support from others

□ Consider career change

□ Other (please specify): ______

□ No symptoms

Q13. What kind of psychological distress did you experience? (Multiple selections possible)

□ Anxiety

□ Depression

□ Lethargy

□ Recall of the situation at the workplace

□ Recall of the situation outside the workplace

□ Other (please specify): ______

□ No symptoms

Q14. What kind of physical distress did you experience? (Multiple selections possible)

□ Loss of sleep

□ Headache

□ Back pain

□ Fatigue

□ Loss of appetite

□ Nausea

□ Nightmare

□ Other (please specify): ______

□ No symptoms

Q15. How did it affect your own behavior?

□ Lack of concentration

□ Defensive, overprotective behavior

□ Aggressive, risky behavior

□ Use of alcohol or other substances

□ Loss of interest in daily activities

□ Other (please specify): ______

□ No symptoms

Q16. Recover time from symptoms:

□ ≤ 1 day

□ ≤ 1 week

□ ≤ 1 month

□ ≤ 1 year

□ > 1 year

□ > 1 year, symptoms remain

□ No symptoms

Q17. Impact on work:

□ Overcame and used the experience to improve

□ Continued to work, no symptoms

□ Continued to work, but with symptoms

□ Left work

Q18. After the patient safety incident, who did you ask for support?

□ Colleague

□ Supervisor

□ Consultant within an institution

□ Friend

□ Family

□ Other (please specify): ______

□ No one

Section D. About the patient safety incident.

We will ask you about the patient safety incident you recall.

Q19. How long ago did the incident occur?

□ Within 1 month

□ Within 1 to 3 months

□ Within 3 to 6 months

□ Within 6 months to 1 year

□ Within 1 to 3 years

□ More than 3 years ago

Q20. What kind of patient safety incident was it?

□ Diagnosis-related

□ Medication-related

□ Patient care-related

□ Procedure-related

□ Surgery-related

□ Infection-related

□ Other (please specify): ______

Q21. Were there any errors or harm to patients in that incident?

□ There were errors, but they were prevented.

□ There were errors, but there was no harm to patients.

□ There were errors and harm to patients.

□ There were no errors, but there was harm to patients.

Q22. Severity of harm caused by the incident:

□ No harm

□ Temporary harm, required intervention

□ Temporary harm, required initial or prolonged hospitalization

□ Permanent patient harm

□ Required intervention necessary to sustain life

□ Patient’s death

Items Q23–Q29 were adapted from the Japanese version of the Agency of Healthcare Research and Quality’s Hospital Survey on Patient Safety Culture.

Items Q30–Q71 were adapted from the Japanese version of Second Victim Experience and Support Tool – Revised.

Q72. Please feel free to write any comments, opinions, or impressions you may have: ______

Appendix 2. Mailing list used for recruitment

| Mailing list | Number of subscribers  N = 18,266 | Number of respondents  N = 884 (%) |
| --- | --- | --- |
| Japanese Society for Quality and Safety in Healthcare | 3,435 | 420 (12.2) |
| Japan Society of Clinical Safety | 1,482 | 91 (6.1) |
| Japanese Society of Intensive Care Medicine | 11,968 | 282 (2.4) |
| Nursing committee of Japanese Society of Education for Physicians and Trainees in Intensive Care | 679 | 30 (4.4) |
| Medical Safety Conference (Patient Safety Unit of Kyoto University Hospital) | 647 | 54 (8.3) |
| Japanese Red Cross Society Pharmaceutical Association | 55 | 7 (12.7) |

Appendix 3. Department in which PSIs occurred

| Location | n = 884 (%) |
| --- | --- |
| Surgical ward | 172 (19.5) |
| Internal medicine ward | 145 (16.4) |
| Intensive care unit | 159 (18.0) |
| Operating room | 113 (12.8) |
| High care unit | 38 (4.3) |
| Emergency department | 34 (3.8) |
| Pediatrics | 32 (3.6) |
| Pediatric Intensive Care Unit | 17 (1.9) |
| Neonatal Intensive Care Unit | 7 (0.8) |
| Obstetrics and Gynecology | 24 (2.7) |
| Outpatient department | 21 (2.4) |
| Anesthesiology | 20 (2.3) |
| Radiology | 11 (1.2) |
| Rehabilitation | 9 (1.0) |
| Pharmacy | 27 (3.1) |
| Psychiatry | 7 (0.8) |
| Laboratory | 17 (1.9) |
| Dialysis Room | 7 (0.8) |
| Administration | 7 (0.8) |
| Others | 17 (1.9) |

Abbreviation: PSI = patient safety incident.

Appendix 4. Spearman’s correlation matrix between variables used in the SEM analysis

|  | Female | Year of experience ≤ 5 | Harm severity | Teamwork within units | Nonpunitive response to errors | Psychological distress | Physical distress | Professional Self-Efficacy | Colleague Support | Supervisor Support | Institutional Support | Turnover Intentions | Absenteeism | Resilience |
| --- | --- | --- | --- | --- | --- | --- | --- | --- | --- | --- | --- | --- | --- | --- |
| Female | 1.000 |  |  |  |  |  |  |  |  |  |  |  |  |  |
| Year of experience ≤ 5 | 0.051 | 1.000 |  |  |  |  |  |  |  |  |  |  |  |  |
| Harm severity | -0.077* | -0.193** | 1.000 |  |  |  |  |  |  |  |  |  |  |  |
| Teamwork | 0.054 | -0.068* | 0.076* | 1.000 |  |  |  |  |  |  |  |  |  |  |
| Nonpunitive response | -0.087* | -0.123** | 0.084* | 0.350** | 1.000 |  |  |  |  |  |  |  |  |  |
| Psychological distress | 0.128** | 0.123** | -0.067* | -0.173** | -0.348** | 1.000 |  |  |  |  |  |  |  |  |
| Physical distress | 0.115** | 0.043 | 0.046 | -0.180** | -0.293** | 0.635** | 1.000 |  |  |  |  |  |  |  |
| Professional Self-Efficacy | -0.018 | 0.065 | -0.076* | -0.475** | -0.422** | 0.464** | 0.432** | 1.000 |  |  |  |  |  |  |
| Colleague Support | 0.046 | 0.019 | -0.056 | -0.449** | -0.440** | 0.241** | 0.291** | 0.579** | 1.000 |  |  |  |  |  |
| Supervisor Support | 0.010 | 0.060 | -0.030 | -0.376** | -0.349** | 0.257** | 0.225** | 0.383** | 0.493** | 1.000 |  |  |  |  |
| Institutional Support | 0.147** | 0.156** | -.077* | -0.180** | -0.303** | 0.745** | 0.625** | 0.502** | 0.229** | 0.204** | 1.000 |  |  |  |
| Turnover Intentions | 0.164** | 0.091** | -0.038 | -0.247** | -0.355** | 0.603** | 0.661** | 0.571** | 0.378** | 0.251** | .649** | 1.000 |  |  |
| Absenteeism | 0.052 | 0.064 | -0.037 | -0.170** | -0.219** | 0.304** | 0.472** | 0.406** | 0.237** | 0.055 | .388** | 0.497** | 1.000 |  |
| Resilience | -0.037 | -0.015 | -0.054 | -0.247** | -0.160** | 0.081* | 0.156** | 0.391** | 0.380** | 0.262** | .168** | 0.314** | 0.226** | 1.000 |

**p* < 0.05; ***p* < 0.01

Abbreviation: SEM = structure equation modeling.

Appendix 5. SEM for involvement group in PSIs showing the associations between organizational support, SV distress, and outcomes


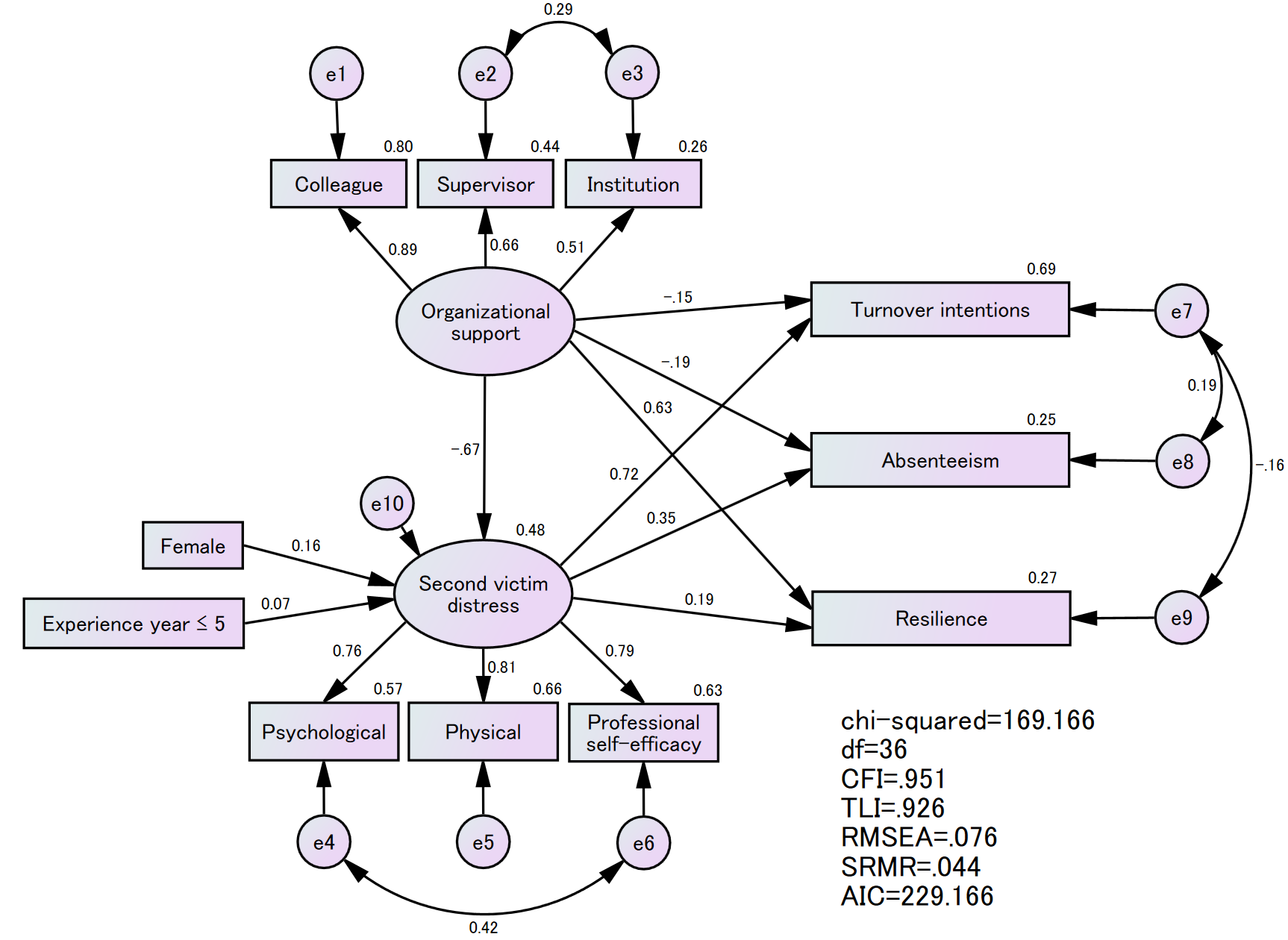


Abbreviation: PSI = patient safety incident; SEM = structure equation modeling; SV = second victim.

Appendix 6. SEM for involvement group in PSIs showing the associations between patient safety culture, SV distress, and outcomes


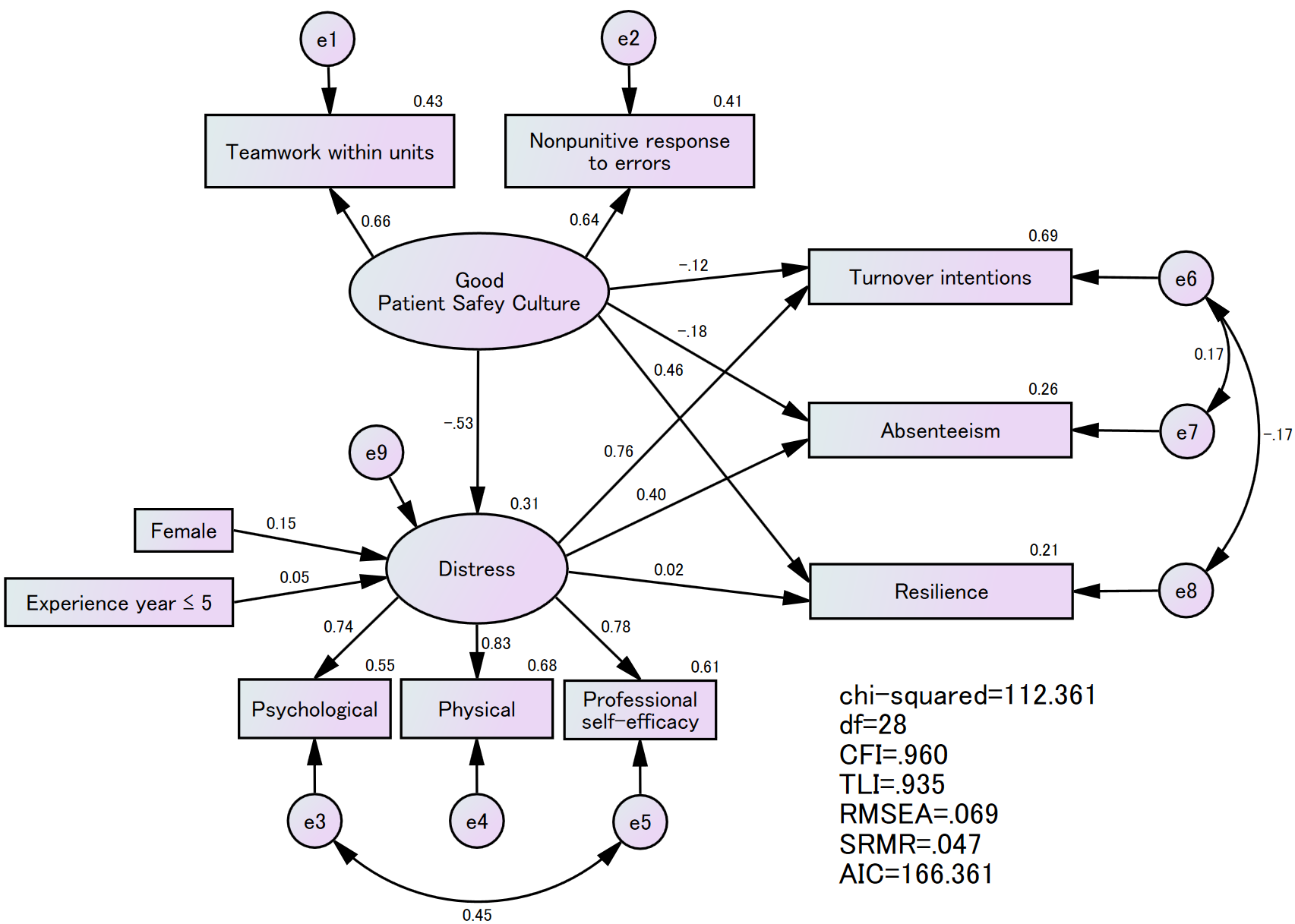


Abbreviation: PSI = patient safety incident; SEM = structure equation modeling; SV = second victim.

Appendix 7. Comprehensive graphical summary of the JaSeVic study findings.


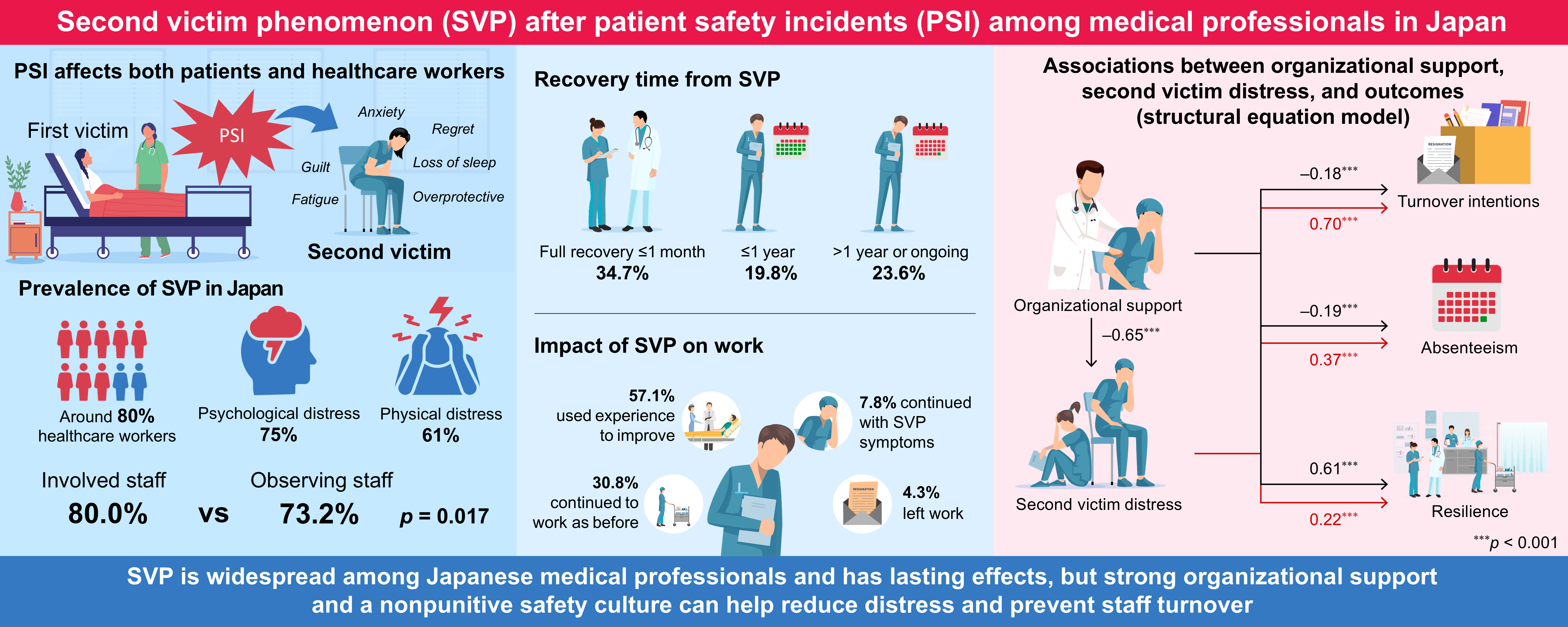

Supplement: Supplementary file 1 — Supplementary Material 1 [file 12913_2026_14251_MOESM1_ESM.docx]
